# Nifuroxazide ameliorates lipid and glucose metabolism in palmitate-induced HepG2 cells

**DOI:** 10.1039/c9ra06527j

**Published:** 2019-11-29

**Authors:** Jing-Yi Liu, Yi-Chen Zhang, Li-Ni Song, Lin Zhang, Fang-Yuan Yang, Xiao-Rong Zhu, Zhi-Qiang Cheng, Xi Cao, Jin-Kui Yang

**Affiliations:** Beijing Diabetes Institute, Beijing Key Laboratory of Diabetes Research and Care, Department of Endocrinology, Beijing Tongren Hospital, Capital Medical University Beijing 100730 China jinkui.yang@gmail.com tide005@126.com +86-10-58268445; Department of Pharmacology and Molecular Sciences, Johns Hopkins University School of Medicine Baltimore MD 21205 USA

## Abstract

Inflammation constitutes an important component of non-alcoholic fatty liver disease. STAT3 is a direct target of inflammatory cytokines, but also mediates glycolipid metabolism in the liver. As a potent inhibitor of STAT3, the effect of Nifuroxazide (Nifu) on glycolipid metabolism in liver has not been reported. In this study, we used palmitic acid (PA)-induced HepG2 cells to examine the expression of inflammatory factors and apoptosis-related proteins and the content of triglyceride (TG), total cholesterol (TC), and glycogen. The expression of hepatic lipogenic proteins (ACCα, SREBP-1c, FAS), gluconeogenesis enzymes (PEPCK, G6Pase, and IRS2), the IL-6/STAT3/SOCS3 inflammatory axis, and the insulin signaling pathway was determined. Our study shows that Nifu significantly improves lipid metabolism disorders in the PA-induced HepG2 cells, whereas, it remarkably reduced intracellular free fatty acid (FFA), TG, and TC content, suppressed lipid synthesis, and increased lipid decomposition. Our results also showed that Nifu significantly improved dysregulated glucose metabolism in the PA-treated HepG2 cells, increased glycogen content, and inhibited gluconeogenesis. Further research indicated that Nifu markedly inhibited activation of the IL-6/STAT3/SOCS3 signaling pathway. Finally, due to anti-inflammatory stress, Nifu enhanced insulin signaling in the PA-induced HepG2 cells. Therefore, Nifu can improve glucose and lipid metabolism in the PA-induced HepG2 cells, which provides new evidence that Nifu has a positive effect on PA-induced cellular hepatic steatosis and improves glucose metabolism in HepG2 cells, providing a new perspective for studying drug treatment of glucose and lipid metabolism disorders.

## Abstract 2

Inflammation constitutes an important component of non-alcoholic fatty liver disease.

## Graphical abstract

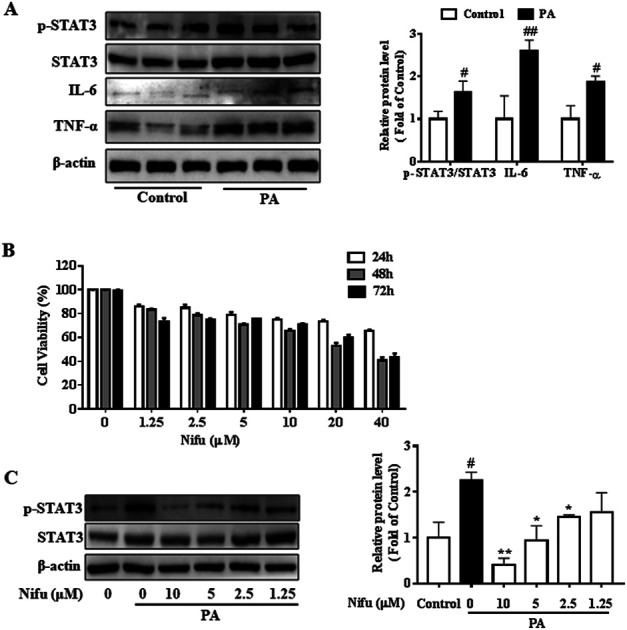

## Introduction

Non-alcoholic fatty liver disease (NAFLD), one of the most common liver diseases, is characterized by accumulation of triglycerides (TG) in liver cells, which increases the risk of diabetes and cardiovascular disease.^1^ According to the “two-hit” hypothesis, hepatic cell lipid accumulation is caused by insulin resistance to comprise the first hit for NAFLD. Further, an inflammatory response, oxidative stress, and lipid peroxidation exacerbate hepatocyte injury become the second hit of NAFLD, causing inflammatory damage, necrosis, and fibrosis in the liver.^2^ Therefore, inflammatory responses, insulin resistance, and lipid metabolic disorders represent an important component of the “two-hit” hypothesis of NAFLD.

Recently much evidence has suggested that chronic low-grade inflammation plays a central role in glucose and lipid metabolic disorders. The accumulation of inflammatory factors, excessive free fatty acid (FFA) influx, and increased lipid intermediates in the liver can impair the homeostasis of glucose and lipid metabolism and accelerate the progression of hepatic insulin resistance, type 2 diabetes mellitus (T2DM), and NAFLD.^3,4^ Various proinflammatory mediators such as tumor necrosis factor-α (TNF-α), interleukin (IL)-6, IL-1β, and cyclooxygenase-2 are increased in the livers of NAFLD patients.^5^ IL-6 is an important inflammatory cytokine and increased circulating levels often result in deleterious metabolic behavior, causing obesity, diabetes, and NAFLD.^6^

Signal transducer and activator of transcription 3 (STAT3), as a direct target of IL-6, plays an important role in glucose and lipid metabolism in the liver.^7,8^ IL-6 can promote STAT3 phosphorylation and activation, causing insulin resistance and dyslipidemia.^9,10^ Further, overexpression of the active form of STAT3 in the liver inhibits hepatic glucose production and significantly improves glucose intolerance.^11^ Studies have also shown that its inhibition can reduce the expression of high glucose-induced lipogenic genes, the TG content, and cholesterol levels.^12,13^ In addition, activated STAT3 regulates the expression of genes involved in insulin sensitivity and glycolipid metabolism by increasing SOCS3 levels. In contrast, the STAT3 signaling also stimulates the expression of lipogenic genes and promotes the *de novo* synthesis of fatty acids by increasing the expression of hepatic olfactory regulatory element binding protein-1c (SREBP-1c).^14^ Finally, STAT3 signaling increases hepatic gluconeogenesis by increasing the expression of key enzymes involved in gluconeogenesis and reduces insulin sensitivity by inhibiting the phosphorylation of PI3K and AKT in the liver.^15^ Therefore, this signaling molecule could be an important therapeutic target for glucose and lipid metabolic disorders.

Nifu (4-hydroxy-*N*-[(*E*)-(5-nitrofuran-2-yl) methylideneamino] benzamide), an oral nitrofuran antibiotic, was shown to be a potent and effective inhibitor of STAT3. By inhibiting the phosphorylation of this protein, it exerts multiple biological effects, including anti-inflammatory activity, exploited treat acute diarrhea^16,17^ and anti-cancer effects;^18^ it can also delay the development of diabetic nephropathy.^19^ However, it is unknown if Nifu has therapeutic effects on glucose and lipid metabolic disorders. In view of the positive effects of Nifu on biological processes and STAT3, as well as the important role of this signaling molecule in NAFLD and hepatic insulin resistance, we assessed whether Nifu could attenuate hepatic glycolipid metabolic disorders and mitigate liver inflammation by inhibiting the STAT3 cascade using a palmitic acid (PA)-induced HepG2 cell model.

## Materials and methods

### Cell culture and treatments

Human hepatoma (HepG2 line) (Cell Resource Center, IBMS, CAMS/PUM) cells were maintained in DMEM (Corning, Germany) with 10% fetal bovine serum (Hyclone, USA), 100 units per mL penicillin, and 0.1 mg mL^−1^ streptomycin. Cells were cultured at 37 °C with humidified air and 5% CO_2_. Trypsinization was used to passage the cells every 2 days. HepG2^105^ was used to subsequent experiments. To establish a model of hepatocellular injury, the HepG2 cells were pre-treated with 0.2 mM PA (Sigma-Aldrich, Canada) for 24 h. Nifu was purchased from Xiyashiji Chemical Co. (ChengDu, Sichuan, China). Purity (98%) was measured by high-performance liquid chromatograph analysis. Nifu was dissolved to produce a 20 mM stock solution in dimethyl sulfoxide (DMSO) and diluted to the indicated concentrations in DMEM for a 48 h treatment. The equivalent amount of DMSO was diluted in the medium of control group.

### Cell viability assay

The viability of the Nifu-treated HepG2 cells was assessed by performing MTT assays (Sigma-Aldrich, Canada). The HepG2 cells were cultured in 96-well plates (7 × 10^3^ per well) with gradient concentrations of Nifu for 24, 48, and 72 h. After the treatment, 20 μL of 5 mg mL^−1^ MTT was added to each well and cells were cultured for 4 h at 37 °C. Then the medium was removed, and insoluble formazan crystals were dissolved in DMSO. The color absorbance was measured at 490 nm using a microplate reader (Bio-Rad, USA).

### Oil Red O staining

The HepG2 cells were seeded in 6-well plates and stained with a commercial Oil Red O staining kit (Solarbio, China). The HepG2 cells were washed with phosphate buffered saline (PBS) and fixed with ORO Fixative for 1 h. Cells were rinsed with ddH_2_O, dipped in 60% isopropanol for 5 min, stained with the ORO stain for 20 min, and rinsed with ddH_2_O 2–5 times to remove excess staining solution. The nuclei were stained with Mayer hematoxylin staining solution for 1–2 min and washed 2–5 times. ORO buffer was added for 1 min and finally, distilled water was added to cover the cells, which were observed under a microscope (Bio-Rad, USA). The Oil Red O staining was quantified based on the OD value at 490 nm using a microplate reader (Bio-Rad, USA).

### Glycogen assay

According to Glycogen assay kit instructions (Biovision, USA), 1 × 10^6^ HepG2 cells were resuspended in 200 μL dH_2_O on ice. The homogenates were boiled for 10 min and centrifuged at 18 000 × *g* for 10 min. The supernatant was collected and tested; specifically, 30 μL samples were added to a 96-well plate and hydrolysis buffer was used to adjust the final volume to 50 μL. Then, the plates were added 2 μL hydrolysis enzyme mixture per well and incubated for 30 min at room temperature. Afterwards, 50 μL of reaction mix was added to each well and incubated for 30 min at room temperature. The absorbance was measured at 570 nm using a microplate reader (Bio-Rad, USA).

### Measurement of triglycerides (TG) and total cholesterol (TC)

Quantitative analysis of TG and TC in the cells were carried out by an enzymatic assay kit (Applygen, China). 0.1 mL of the lysate was added to 1 × 10^6^ cells, and the mixture was incubated at room temperature for 10 min. The supernatant was then transferred to two new centrifuge tubes. One was used for enzymatic determination by the supernatant collected from the sample after heated at 70 °C for 10 min and then centrifuged at 2000 rpm for 5 min. Another one was for protein quantification. The protein concentration was assessed using a bicinchoninic acid (BCA) Protein Assay Kit (Beyotime Biotechnology, China). All samples were tested in duplicate, and the intracellular TG and TC levels were normalized to the protein concentration.

### Measurement of free fatty acid

Free Fatty Acid Quantification Assay Kit (Colorimetric/Fluorometric) was used (Abcam, UK). The collection and preparation of cell samples were performed according to kit instructions. Specifically, 1 × 10^6^ cells were mixed with 200 μL chloroform/Triton X-100 (1% Triton X-100 in pure chloroform) by pipetting up and down and then incubated on ice for 10–30 min. Next, the extract was centrifuged for 5–10 min to collect the lower phase, which was then air dried at 50 °C in a fume hood to remove chloroform. The dried lipids were dissolved in 200 μL of Fatty Acid Assay Buffer for 5 min. Finally, 50 μL of reaction mix was added to each well and incubated at room temperature for 30 min. The result was recorded using a microplate reader at 570 nm (Bio-Rad, Hercules, CA, USA).

### Quantitative real-time PCR (qRT-PCR)

500 ng RNA from the HepG2 cells was isolated with an RNAprep pure tissue kit and RNAprep pure cell/bacteria kit (Tiangen Biotech, Beijing, China) and used to synthesize first-strand cDNA using the TransScript First-Strand cDNA Synthesis SuperMix kit (Transgen Biotech). cDNA was amplified using TransStart Top Green qPCR SuperMix (Transgen Biotech) and the amplification procedure was performed with a Light Cycler 480 Real-Time PCR system (Roche, Basel, Switzerland). The gene expression level was calculated based on the ΔΔ*C*_q_ method and normalized to β-actin levels for each sample. The RT-PCR-specific primers used were listed in Table 1.

**Table 1:** The RT-PCR-specific primers

Genes	Forward primer	Reverse primer
*ACCα*	ATGTCTGGCTTGCACCTAGTA	CCCCAAAGCGAGTAACAAATTCT
*PPARγ*	CTTGTGAAGGATGCAAGGGT	ATACAAATGCTTTGCCAGGG
*SREBP-1C*	GGAGCCATGGATTGCACATT	GGCCCGGGAAGTCACTGT
*FAS*	TCTGGTTCTTACGTCTGTTGC	CTGTGCAGTCCCTAGCTTTCC
*ACOX-1*	ACTCGCAGCCAGCGTTATG	AGGGTCAGCGATGCCAAAC
*MCAD*	GGGTGGTAGACGCTACAACC	GTGCCCTCAAAACCTGGGTAT
*PPARα*	TCCTGAGCCATGCAGAATTTAC	AGTCTAAGGCCTCGCTGGTG
*CPT-1α*	TCCAGTTGGCTTATCGTGGTG	TCCAGAGTCCGATTGATTTTTGC
*G6pase*	GTGTCCGTGATCGCAGACC	GACGAGGTTGAGCCAGTCTC
*PEPCK*	GCCATCATGCCGTAGCATC	AGCCTCAGTTCCATCACAGAT
*Glut2*	GCTGCTCAACTAATCACCATGC	TGGTCCCAATTTTGAAAACCCC
*IRS2*	CGGTGAGTTCTACGGGTACAT	TCAGGGTGTATTCATCCAGCG
*IL-6*	ACTCACCTCTTCAGAACGAATTG	CCATCTTTGGAAGGTTCAGGTTG
*TNF-α*	CCTCTCTCTAATCAGCCCTCTG	GAGGACCTGGGAGTAGATGAG
*MCP-1*	CAGCCAGATGCAATCAATGCC	TGGAATCCTGAACCCACTTCT
*STAT3*	CAGCAGCTTGACACACGGTA	AAACACCAAAGTGGCATGTGA
*SOCS3*	CCTGCGCCTCAAGACCTTC	GTCACTGCGCTCCAGTAGAA
*β-actin*	GTGACGTTGACATCCGTAAAGA	GCCGGACTCATCGTACTCC

### Western blot analysis

The HepG2 cells were collected and washed twice with PBS; then, the cells were lysed in RadioImmunoprecipitation Assay (RIPA) buffer containing 100 mmol L^−1^ phenylmethylsulfonyl fluoride and 25 mmol L^−1^ proteinase inhibitor (Roche, Switzerland). The total protein concentration was assessed using a BCA Protein Assay Kit (Beyotime Biotechnology, China). Approximately 40 μg of sample protein was separated using 10% Mini-RROTEAN TGX SDS-Stain-Free Precast gels (Bio-Rad, USA) and then transferred to polyvinylidene fluoride membranes (Millipore, USA). After transfer, the membrane was blocked with 5% non-fat dry milk at room temperature. Specific primary antibodies were incubated with the blot at 4 °C overnight. The signal was detected with HRP-conjugated anti-IgG followed by detection with ECL (Millipore) and the images were scanned using a ChemiDoc Touch Imaging System (Bio-Rad). Antibodies against STAT3, phospho-STAT3, IL-6, TNF-α, G6pase, PEPCK, IRS2, Glut2, FAS, CPT-1α, IRS-1, phospho-IRS-1(Ser307), AKT, phospho-AKT (Ser473), FOXO1, phospho-FOXO1 (Ser256), Caspase3 and β-actin were purchased from Cell Signaling Technology (USA). Antibodies against SREBP-1C and ACCα were purchased from Santa Cruz Biotechnology (USA). Antibodies against Bax and Bcl2 were purchased from Abcam (UK).

### Statistics analysis

All results are expressed as the mean ± SEM and analysis was performed with the statistical software GraphPad Prism version 7.0. A Student's *t*-test or one-way ANOVA test with a Dunnett's multiple comparison test was used to analyze two sets of independent data that conform to a normal distribution and have a uniform variance, and *p* < 0.05 was considered statistically significant.

## Results

### Effects of Nifu on HepG2 cell viability

**Fig. 1:**
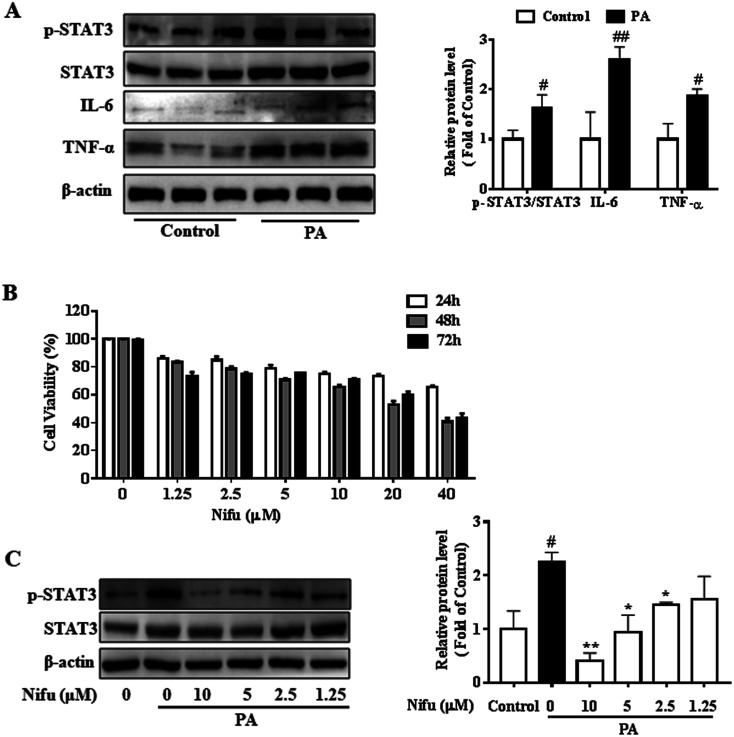
Effects of Nifu on cell viability and the expression of STAT3 in the PA-induced HepG2 cells. (A) The protein expression of inflammatory factors (STAT3, IL-6, TNF-α) in the PA-induced HepG2 cells. (B) Cell viability measurement. 0.2 mM palmitate (PA) induced HepG2 cells for 24 h, and cells were incubated with concentration gradient of Nifu (0, 1.25, 2.5, 5, 10, 20, 40 μM) for 24, 48 or 72 h, respectively, and cell viability was measured by MTT assay. (C) The protein expression of phospho-STAT3/STAT3. Cells were treated with 0.2 mM PA in the absence or presence of Nifu (1.25, 2.5, 5, 10 μM) for 48 h. The data were presented as mean ± SD of three independent experiments. ^#^*P* < 0.05, and ^##^*P* < 0.01 *vs.* vehicle-treated control, *P < 0.05, and **P < 0.01 *vs.* palmitate-treated cells (PA). Control with DMSO is vehicle treated cells.

First, in order to verify the impact of PA on the inflammatory response, we examined the protein expression levels of inflammatory factors in the PA-induced and control-treated HepG2 cells, as shown in Fig. 1A, the expressions of IL-6, TNF-α and phospho-STAT3 in the PA-induced HepG2 cells were significantly higher than those of the control group. The protein levels of inflammatory factors in PA-induced group increased more than twice as much as that in control group. The result indicated PA significantly induced an inflammatory response. Next, to access the effect of Nifu on HepG2 viability, the cells were treated with a concentration gradient (40, 20, 10, 5, 2.5, 1.25 μM) for 24, 48, and 72 h, respectively. As presented in Fig. 1B, based on MTT assay results, no significant changes in cell viability were observed after 48 h of Nifu treatment. Because Nifu was not significantly toxic to cells and the half maximal inhibitory concentration (IC50) was 29.5 μM, cells were treated with 10, 5, 2.5, and 1.25 μM for 48 h in further experiments.

In addition, we used the concentration gradient of Nifu for 48 h to examine the expression of phospho-STAT3, a downstream inflammatory signaling molecule, in the HepG2 cells and found that 5 μM and 2.5 μM exerted a marked inhibitory effect on phospho-STAT3 (Fig. 1C). Therefore, 2.5 and 5 μM (for 48 h) Nifu were used as the optimal concentrations for further experiments.

### Nifu decreases lipid accumulation in PA-induced hepatocyte steatosis

**Fig. 2:**
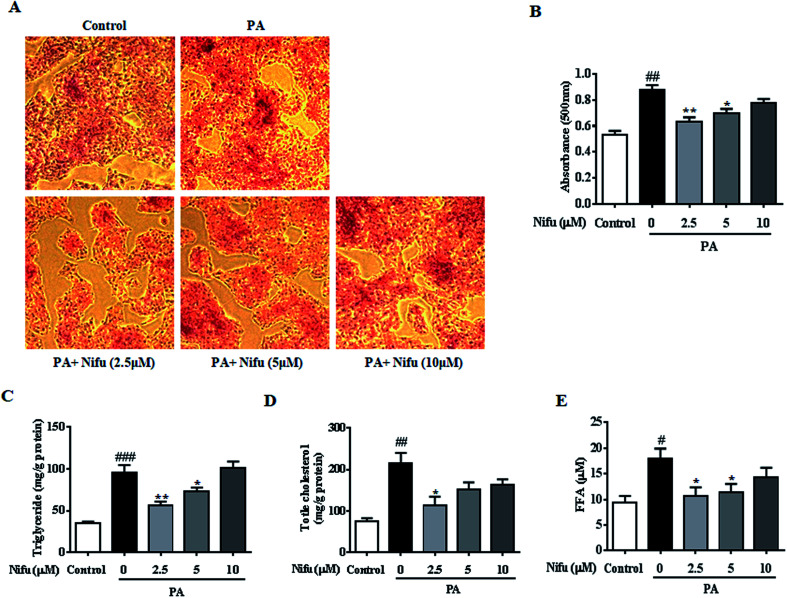
Effects of Nifu attenuated intracellular lipid content in the PA-induced HepG2 cells. (A) Oil Red O staining to assess the cellular lipid contents. (B) Oil Red O staining was quantified by OD value at 490 nm. (C and D) The cellular contents of TG, TC in the PA-induced HepG2 cell, which were treated with various concentrations of Nifu (2.5, 5, 10 μM) for 48 h. (E) The level of FFA. Data were presented as mean ± SEM of three independent experiments. ^#^*P* < 0.05, ^##^*P* < 0.01, and ^###^*P* < 0.001 *vs.* vehicle-treated control; *P < 0.05, and **P < 0.01 *vs.* palmitate-treated cells (PA). Scale bar is 200 μm.

To evaluate the effect of Nifu on lipid accumulation in the PA-induced hepatocyte steatosis, the HepG2 cells were treated with PA for 24 h and then administered 10, 5 and 2.5 μM of Nifu for 48 h. The results of the Oil Red O staining showed the accumulation of red lipid droplets in the 2.5 and 5 μM groups was significantly reduced compared to that in the PA group (Fig. 2A and B). The detection of intracellular TG, TC, and FFA content showed that the expression levels of all three were significantly decreased in the cells treated with 2.5 μM compared to those in the PA group. When cells were treated with 5 μM Nifu, intracellular TG content was also significantly reduced, compared to that in the PA group (Fig. 2C and E). These results indicated that Nifu can markedly suppress intracellular lipid deposition in PA-treated hepatocytes and the concentration of 2.5 μM is the optimal inhibitory concentration.

### Nifu inhibits lipogenesis and promotes lipolysis in the PA-treated HepG2 cells

**Fig. 3:**
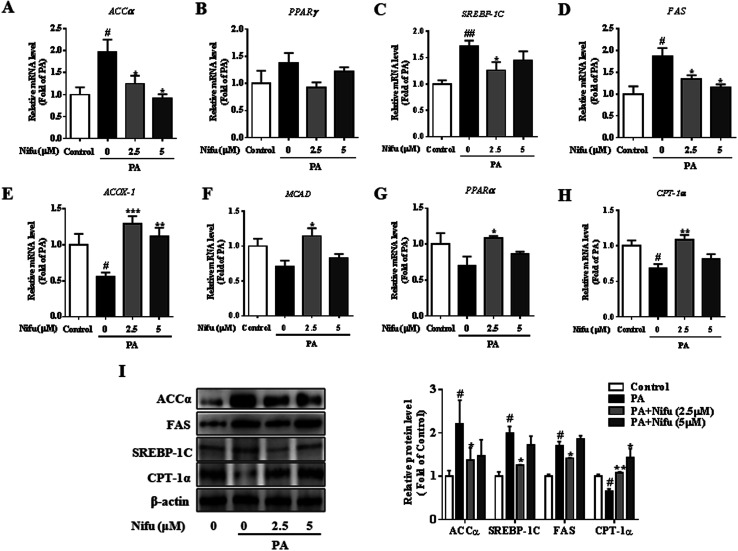
Effects of Nifu inhibited lipogenesis and enhanced lipid oxidation in PA-treated HepG2 cells. (A–D) The mRNA levels of lipid synthesis, including *ACCα*, *PPARγ*, *SREBP-1C* and *FAS*. (E–H) The mRNA levels of key genes of lipid oxidation, including A*COX-1*, *MCAD*, *PPARα* and *CPT-1α*. (I) The expression level of lipid metabolism related proteins. Values were normalized to β-actin. The data were presented as mean ± SEM of three independent experiments. ^#^*P* < 0.05, ^##^*P* < 0.01 *vs.* vehicle-treated control; *P < 0.05, **P < 0.01, and ***P < 0.001 *vs.* palmitate-treated cells (PA).

To further investigate the molecular mechanisms underlying through which Nifu inhibits PA-induced intracellular lipid deposition in the HepG2 cells, mRNA and protein levels of several key enzymes and transcription factors associated with lipid metabolism were detected. As shown in Fig. 3A–D, compared to that in the untreated cells, 2.5 and 5 μM Nifu distinctly suppressed the mRNA expression of most lipogenic genes, including fatty acid synthase (*FAS*), acetyl-coenzyme a carboxylase (*ACCa*), and sterol regulatory element binding protein (*SREBP-1c*). However, the mRNA expression of peroxisome proliferator-activated receptor-γ (*PPARr*) was not significantly different between the treated and untreated groups. This result suggested that Nifu suppresses *de novo* fatty acid synthesis in the HepG2 cells. Quantitative real-time PCR demonstrated that the expression levels of genes related to fatty acid oxidation, including acetyl-CoA oxidase 1 (*Acox1*), peroxisome proliferator-activated receptor alpha (*PPARa*), carnitine palmitoyl transferase 1 (*CPT-1α*), and medium chain acyl-coA dehydrogenase (*MCAD*), were significantly elevated in the 2.5 and 5 μM Nifu groups compared to those in the non-Nifu group (Fig. 3E–H). At the protein level, similar results were obtained by western blotting. Specifically, after treatment with 2.5 μM Nifu, the protein expression levels of ACCα, FAS and SREBP-1C was significantly lower than those in the untreated group, whereas CPT-1α was significantly upregulated compared to PA group after the treatment with 2.5 and 5 μM (Fig. 3I). Therefore, these changes in gene and protein expression indicated that Nifu can suppress lipid synthesis by inhibiting *de novo* fatty acid synthesis and promote lipolysis by inducing fatty acid oxidation in the PA-treated HepG2 cells.

### Effects of Nifu on glucose metabolism in the PA-treated HepG2 cells

**Fig. 4:**
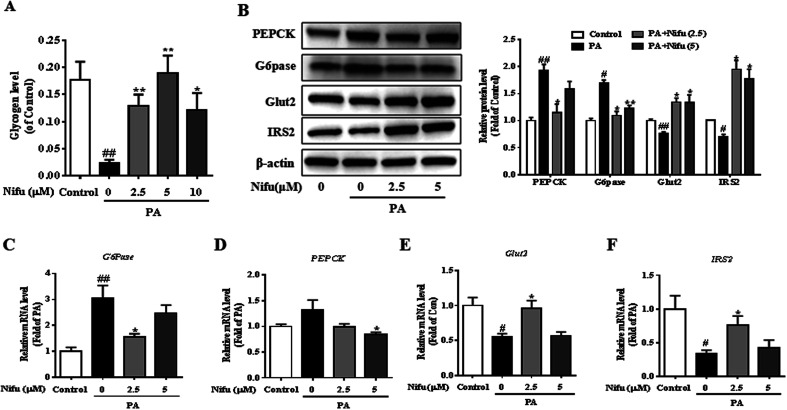
Effects of Nifu improved glucose metabolism in the PA-treated HepG2 cells. (A) The contents of intracellular glycogen synthesis. (B) Relative protein expression levels of PEPCK, G6Pase, IRS2 and Glut2 and densitometric analysis of immunoblot data. Values were normalized to β-actin. The figure showed a representative gel from one experiment. (C–F) Relative mRNA expression levels of *PEPCK*, *G6Pase*, *Glut2* and *IRS2*. The data were presented as mean ± SEM of three independent experiments. ^#^*P* < 0.05, ^##^*P* < 0.01 *vs.* vehicle-treated control; *P < 0.05, and **P < 0.01 *vs.* palmitate-treated cells (PA).

To investigate the effect of Nifu on glucose metabolism in the PA-treated HepG2 cells, we examined intracellular glycogen synthesis. The results showed that the amount of intracellular glycogen synthesis of the PA treated cell was decreased remarkably compared to that in the control cells. Meanwhile, after 2.5 or 5 μM Nifu treatment, intracellular glycogen synthesis was significantly elevated compared with the one in the PA treatment cell (Fig. 4A).

To further explain this finding, the expression levels of genes and proteins involved in glucose metabolism were detected in the PA-treated HepG2 cells. Glucose-6-phosphatase (G6Pase) and phosphoenolpyruvate carboxykinase (PEPCK) are key enzymes in gluconeogenesis in hepatocytes. Insulin receptor substrate 2 (IRS2) is among the important insulin receptor substrates that regulate glucose homeostasis, and glucose transporter 2 (Glut2) is important for hepatocyte glucose transport and uptake and is also the final effector molecule of the insulin signaling pathway. After PA treatment, Nifu inhibited the mRNA and protein expression of G6Pase and PEPCK and enhanced the expression of Glut2 and IRS2 (Fig. 4B–F). These results clearly indicated that Nifu regulates hepatocyte glucose metabolism by suppressing the expression of PEPCK and G6Pase, which inhibits the conversion of non-glucose substrates into glucose, and by inducing Glut2 and IRS2 expression, which enhances the transport of glucose.

### Nifu prevents inflammatory reactions and apoptosis in the PA-treated HepG2 cells

**Fig. 5:**
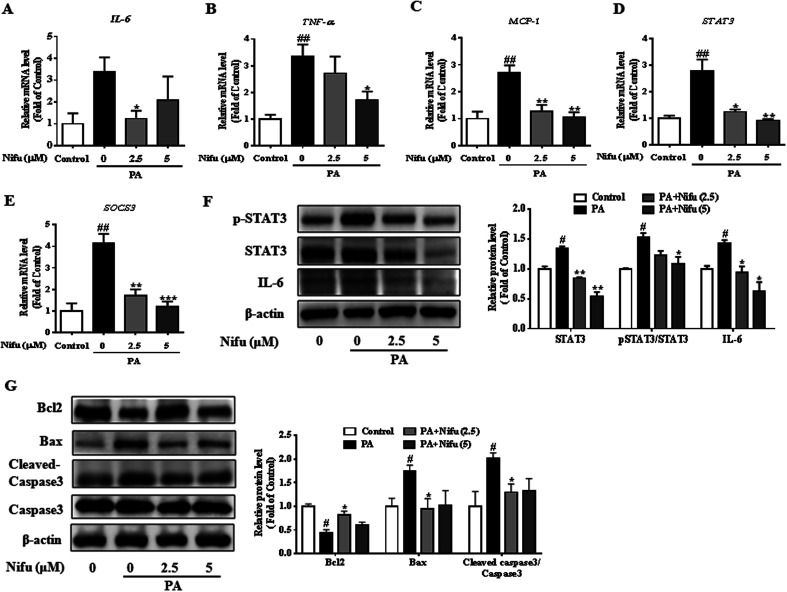
Effects of Nifu suppressed inflammation responses in the PA-treated HepG2 cells. (A–C) The mRNA expression of *IL-6*, *TNF-α*, and *MCP-1*. (D and E) The mRNA expression of *STAT3* and *SOCS3*. (F) The protein expression of IL-6, phospho-STAT3/STAT3 and densitometric analysis of immunoblot data. (G) The protein expression of apoptosis-related protein and densitometric analysis of immunoblot data. Values were normalized to β-actin. The figure showed a representative gel from one experiment. The data were presented as mean ± SEM of three independent experiments. ^#^*P* < 0.05 and ^##^*P* < 0.01 *vs.* vehicle-treated control; *P < 0.05, **P < 0.01, and ***P < 0.001 *vs.* palmitate-treated cells (PA).

Chronic inflammation associated with hepatitis can cause insulin resistance and lipid accumulation in the liver. Recent studies reported that Nifu is an effective inhibitor of STAT3.^20^ To assess the specific effects of Nifu on anti-inflammation, we measured the mRNA levels of inflammatory cytokines including *TNF-α*, *IL-6*, and monocytic chemotactic protein 1 (*MCP-1*). As shown in Fig. 5A–C, after Nifu treatment the mRNA levels of *IL-6* and *TNF-α* in PA-induced HepG2 cells were significantly reduced, respectively, whereas *MCP-1* was significantly downregulated. Additionally, because IL-6 directly activates STAT3, mRNA levels of *STAT3* and cytokine signaling inhibitor 3 (*SOCS3*) were tested to examine the effect of Nifu on the eIL-6/STAT3/SOCS3 signaling. The results showed that PA increased the mRNA expression of *STAT3* and *SOCS3*, which was obviously suppressed by Nifu (Fig. 5D and E). Western blotting analysis further illustrated that PA could increase the protein expression levels of IL-6, phospho-STAT3 and STAT3, and Nifu could decrease IL-6 and phospho-STAT3 levels at all concentrations tested (Fig. 5F).

In addition, apoptosis is closely related to inflammatory response. We detected apoptosis-related proteins and found that B-cell lymphoma-2 (Bcl2) was obviously decreased in PA group. And after treatment with Nifu, the protein expression of Bcl2 was clearly increased in 2.5 μM Nifu group. The protein level of Bax and Cleaved-caspase3 were visibly decreased in 2.5 μM Nifu group compared with the PA group (Fig. 5G). Therefore, combined with the previously mentioned results, we suggest that Nifu has a protective effect on the PA-induced hepatic inflammation.

### Nifu improves PA-induced insulin resistance and insulin signaling

**Fig. 6:**
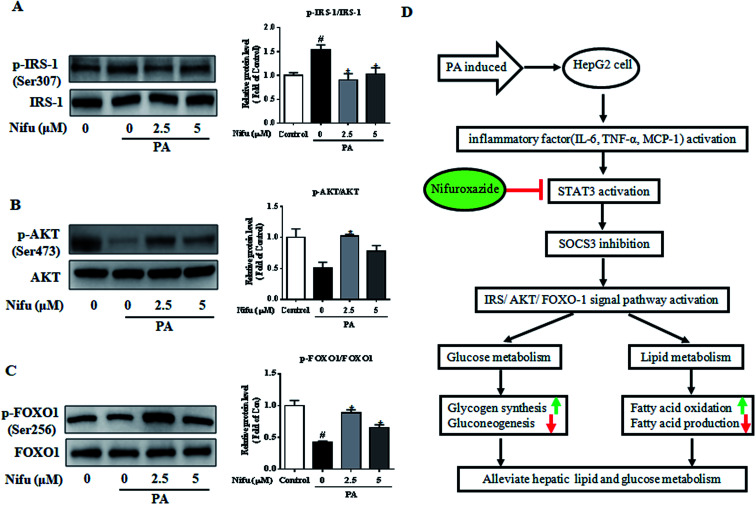
Effects of Nifu enhanced insulin signaling induced in PA-treated HepG2 cells. The phosphorylation and total protein level of (A) IRS-1 (Ser 307), (B) AKT (Ser 473), and (C) FOXO1 (Ser256) were examined by western blot. Left: Image results of western blot. Right: Densitometric analysis of immunoblot data. The figure shows a representative gel from one experiment. Data are exhibited as mean ± SEM of three independent experiments. ^#^*P* < 0.05 *vs.* vehicle-treated control; *P < 0.05 *vs.* palmitate-treated cells (PA). (D) Mechanisms involved in Nifuroxazide ameliorates palmitate-induced lipid and glucose metabolic disorders in the HepG2 cells. The up- or down-regulation of metabolic pathways was indicated by arrows (↑ for upregulation and ↓ for down-regulation).

PA has been shown to impair insulin signaling in hepatocytes^21^ and our finding revealed that Nifu can suppress IL-6/STAT3/SOCS3 signaling in the PA-treated HepG2 cells. To elucidate the mechanism through which Nifu improves PA-induced insulin resistance, we monitored the levels of key proteins involved in the insulin pathway. Fig. 6A–C demonstrated the incubation of PA in HepG2 cells significantly increased the phosphorylation of insulin receptor substrate 1 (IRS-1) (Ser307), compared to that in untreated control cells, which indicated that PA inhibits insulin signaling. However, Nifu noticeably decreased the phosphorylation of IRS-1(Ser307) and clearly increased the protein levels of p-AKT (Ser473) and the phosphorylation of forkhead box protein O1 (p-FOXO1) (Ser256). These data indicate that Nifu effectively increases activation of the insulin signaling pathway.

## Discussion

Patients with NAFLD usually have glucose metabolic disorders and insulin resistance, which not only increases the risk of impaired glucose tolerance, diabetes, or even cardiovascular disease, but also accelerates the progression of NAFLD to non-alcoholic steatohepatitis (NASH), cirrhosis, and even liver cancer.^22^ Further, studies have shown that STAT3 activation can lead to insulin resistance and lipid metabolic disorders.^12^ Therefore, STAT3 might be a potentially important target for disorders associated with glucose and lipid metabolism.

Nifu is a potent inhibitor of STAT3 and has been previously used to treat acute diarrhea.^21^ In recent years, it was also found to be effective against cancer^23^ and to alleviate diabetes-induced kidney damage by inhibiting NF-κB activation, oxidative stress, and apoptosis.^24^ However, no study on its effects on glycolipid metabolism and the related mechanisms was reported. Here, as shown in Fig. 6D, we observed that Nifu inhibits IL-6/STAT3/SOCS3 signaling in hepatocytes, is effective against the expression of inflammatory cytokines, downregulates gluconeogenesis-associated genes and proteins, regulates insulin resistance, downregulates the expression of lipogenesis-associated genes, and suppresses hepatic cell steatosis in the PA-treated HepG2 cells.

Research has shown that increased FFAs, and particularly long-chain saturated fatty acids such as PA, can induce hepatic cell inflammation,^25^ steatosis^26^ and insulin resistance.^27^ Thus, these are widely used to establish hepatic inflammation, steatosis and insulin resistance models with mouse and human liver cell lines.^28^ Fatty acid fluxes in the liver are associated with fatty acid release from adipocytes, which is negatively regulated by insulin; in return, excess glucose in the liver promotes the synthesis of fatty acids and TG.^29^ Generally, fatty acid metabolism in hepatocytes mainly includes the following processes: *de novo* synthesis of fatty acids, uptake of free fatty acids from plasma, and oxidation of fatty acids.^30^ In this study, the mRNA expression of *SREBP-1C*, encoding a chief transcriptional regulator of lipogenesis,^31^ was inhibited and downstream lipogenic rate-limiting enzymes such as FAS and ACCα were also found to be inhibited; these are all key enzymes involved in the *de novo* synthesis of fatty acids.^32^ This result demonstrated that Nifu reduces lipid accumulation in the PA-treated HepG2 cells by inhibiting the *de novo* synthesis of fatty acids. In addition, TG, TC, and FFA levels were significantly reduced in PA-challenged cells after Nifu treatment, which also clearly verified that Nifu can reduce lipid accumulation. In contrast, Nifu potently upregulated the mRNA expression of key genes involved in the oxidative decomposition of fatty acids, such as *CPT-1α*, *PPARα*, *MCAD*, and *ACOX-1*. These results further strongly indicate that Nifu can correct abnormal lipid metabolism in the PA-induced HepG2 cells by suppressing fatty acid synthesis and promoting the oxidative decomposition of fatty acids.

Fatty acids not only cause fat accumulation and inflammatory reaction in liver cells *in vitro* but also induce insulin resistance^33^ and impair glucose transport and glucose metabolism.^34^ In our present research, we discussed that intracellular glycogen synthesis was increased by Nifu in HepG2 cells after PA treatment. In addition, PA treatment was found to result in a significant increase in the protein and mRNA expression of PEPCK and G6Pase, which are key gluconeogenesis enzymes. After Nifu treatment, the protein and mRNA levels of PEPCK and G6Pase decreased significantly. Together, these results illustrate that Nifu inhibits gluconeogenesis and activates glycogen synthesis to prevent PA-induced glucose disorder.

Many studies have shown that inflammation is tightly associated with hepatic steatosis and insulin resistance, simultaneously, PA can reduce hepatic inflammatory reaction^35^ and increased levels of inflammatory cytokines are thought to play an important role in this process.^36^ The mRNA levels of the pro-inflammatory cytokines *IL-6*, *TNF-α*, and *MCP-1* were significantly lower after Nifu treatment in PA-induced HepG2 cells. TNF-α leads to insulin resistance by downregulating IRS-1- and IRS-2-mediated PI3-kinase activation.^37^ IL-6, a major cytokine that regulates insulin resistance, can directly activate the STAT3–SOCS3 pathway in the liver.^38^ Besides, IL-6 upregulates the expression of phospho-STAT3 and then increases the expression of SOCS3 *via* a negative feedback, while SOCS3 disrupts insulin signaling through the ubiquitin-mediated degradation of IRS.^39,40^ Furthermore, apoptosis can regulate the development of inflammatory response to prevent cell or organ damage in the inflammatory response.^41^ In the present study, Nifu was found to obviously inhibit the expression of inflammatory factors and apoptosis-related proteins and vastly mitigate dysregulation of the insulin signaling pathway by regulating IL-6/STAT3/SOCS3 signaling in the PA-treated hepatic cells.

The IRS-1/AKT/FOXO1 signaling cascade controls hepatic glucose and insulin metabolism by modulating multiple genes in the metabolic pathway.^42^ Insulin binds the insulin receptor to activate IRS-1 phosphorylation and activated IRS1 binds the regulatory subunit P85 of phosphatidylinositol 3-kinase (PI3K) to transmit the signal.^43^ Activation of PI3K generates phosphatidylinositol (3,4,5)-triphosphate (PIP3), which activates protein kinase AKT (also called PKB).^44^ Forkhead homeobox class O protein (FOXO1), a substrate for AKT in hepatocytes, regulates a variety of metabolic activities including the regulation of insulin sensitivity,^45^ gluconeogenesis,^46^ and myocardial growth.^47^ Our present study indicated that phosphorylated IRS-1(Ser307) levels were significantly increased in the PA-treated HepG2 cells, whereas they were sharply inhibited after treatment with Nifu, demonstrating that PA induces insulin resistance, whereas Nifu can reverse this. In addition, the insulin signaling pathway (IRS-1/AKT/FOXO1) was activated, further demonstrating that Nifu mitigates PA-induced insulin resistance by activating insulin signaling.

We noticed this study had some limitations. First, our work was carried out using a single cell line *in vitro*, which cannot mimic the physiological state of the body. To further clarify the mechanism underlying the effects of Nifu, a high-fat-diet obese mouse model or db/db mice should be used to determine the integrated effect of Nifu on body weight, lipid metabolism, and glucose metabolism. In addition, considering the anti-inflammatory effects of the drug itself, it is necessary to further determine whether it affects the intestinal flora, and thus modulates energy metabolism, by performing animal studies.

Although the causes and underlying mechanisms of NAFLD and glucose metabolism disorder require further exploration, it clears that Nifu can normalize the inflammatory response and improve liver glycolipid metabolic disorders. There is no doubt that Nifu might represent a new strategy for the treatment or prevention of glucose and lipid metabolic disorders in liver.

## Conclusions

In this study, we used a cell model of non-alcoholic fatty liver and insulin resistance, namely PA-induced HepG2 cells, to prove the following finding for the first time: (1) Nifu decreases liver lipid accumulation and protects against NAFLD by enhancing fatty acid oxidation and reducing fatty acid production; (2) Nifu regulates glucose metabolism by inhibiting gluconeogenesis and activating glycogen synthesis; (3) Nifu alleviates PA-induced inflammatory responses and insulin resistance by modulating STAT3 signaling and the IL-6/STAT3/SOCS3 pathway.

## Author contributions

Jing-Yi Liu contributed to developing research methodology, analysis, and writing the manuscript. Yi-Chen Zhang, Li-Ni Song, Lin Zhang, Fang-Yuan Yang, Xiao-Rong Zhu coordinated to collect and analyze research data. Zhi-Qiang Cheng coordinated to revise the manuscript. Xi Cao and Jin-Kui Yang contributed to the design of the study, research data analysis, and wrote manuscript, and coordinated submission.

## Conflicts of interest

The authors declare no conflict of interest.

## Supplementary Material
